# Cardiac Cyclic Nucleotide Phosphodiesterases: Roles and Therapeutic Potential in Heart Failure

**DOI:** 10.1007/s10557-020-06959-1

**Published:** 2020-03-14

**Authors:** Michael E. J. Preedy

**Affiliations:** William Harvey Research Institute, Barts & The London School of Medicine & Dentistry, Queen Mary University of London, Charterhouse Square, London, EC1M 6BQ UK

**Keywords:** Cyclic AMP, Cyclic GMP, Heart failure, Phosphodiesterase

## Abstract

The cyclic nucleotides cyclic adenosine-3′,5′-monophosphate (cAMP) and cyclic guanosine-3′,5′-monophosphate (cGMP) maintain physiological cardiac contractility and integrity. Cyclic nucleotide–hydrolysing phosphodiesterases (PDEs) are the prime regulators of cAMP and cGMP signalling in the heart. During heart failure (HF), the expression and activity of multiple PDEs are altered, which disrupt cyclic nucleotide levels and promote cardiac dysfunction. Given that the morbidity and mortality associated with HF are extremely high, novel therapies are urgently needed. Herein, the role of PDEs in HF pathophysiology and their therapeutic potential is reviewed. Attention is given to PDEs 1–5, and other PDEs are briefly considered. After assessing the role of each PDE in cardiac physiology, the evidence from pre-clinical models and patients that altered PDE signalling contributes to the HF phenotype is examined. The potential of pharmacologically harnessing PDEs for therapeutic gain is considered.

## Introduction

Cyclic adenosine-3′,5′-monophosphate (cAMP) and cyclic guanosine-3′,5′-monophosphate (cGMP) are cyclic nucleotides that serve as key second messengers within the heart. As siblings, cAMP and cGMP concurrently preserve cardiac physiology despite frequently signalling in a contrary fashion. The cyclic nucleotide phosphodiesterases (PDEs) facilitate cyclic nucleotide degradation by catalysing the hydrolysis of the phosphodiester bond of cAMP and/or cGMP. Reducing, both spatially and temporally, the intracellular concentration of cyclic nucleotides, PDEs compartmentalise cAMP and cGMP signalling and are vital for mediating crosstalk between the two pathways. The expression and/or activity of multiple PDEs is altered during heart failure (HF). HF affects approximately 37 million people worldwide and is typified by a chronic deterioration of cardiac function during which the heart is unable to sustain a cardiac output (CO) sufficient for the maintenance of tissue homeostasis [1, 2]. Given the influence of cAMP and cGMP on cardiac contractility, hypertrophy, fibrosis and apoptosis, variations in PDE and cyclic nucleotide levels contribute to the cardiac dysfunction characteristic of HF. Understanding the role of PDEs in HF pathophysiology may identify novel targets for pharmacological manipulation and thus tackle the existing shortfall of effective therapies. Considering that nearly half of all HF patients die within 5 years of diagnosis [3, 4], this is certainly a pressing aim.

## Cyclic Nucleotides in Cardiac Physiology

**Fig. 1:**
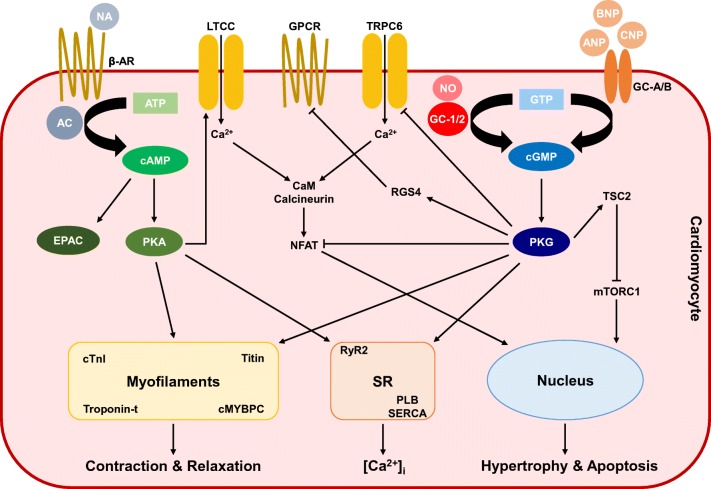
Cyclic nucleotide signalling within cardiomyocytes. Cyclic adenosine-3′,5′-monophosphate (cAMP) is synthesised from adenosine-5′-triphosphate (ATP) by the action of adenylyl cyclases (ACs) in response to noradrenaline (NA). Cyclic guanosine-3′,5′-monophosphate (cGMP) is synthesised from guanosine-5′-triphosphate (GTP) by the action of guanylyl cyclases (GCs). GC-1/2 is stimulated by nitric oxide (NO) and is primarily located within the cytosol. Transmembrane GC-A is activated by atrial natriuretic peptide (ANP) and brain natriuretic peptide (BNP), and GC-B is triggered by C-type natriuretic peptide (CNP). cAMP and cGMP act predominantly via activation of protein kinase A (PKA) and protein kinase G (PKG), respectfully. cAMP also binds exchange protein activated by cAMP (EPAC). Both PKA and PKG phosphorylate multiple cardiomyocyte proteins to effect changes in chronotropy, inotropy and lusitropy, as well as cardiomyocyte hypertrophy and apoptosis. Arrows indicate stimulation. Blunt lines indicate inhibition. Abbreviations: [Ca^2+^]_i_, intracellular concentration of calcium; β-AR, beta-adrenergic receptor; Ca^2+^, calcium; CaM, calmodulin; cMYBPC, cardiac myosin–binding protein C; cTnI, cardiac troponin I; GPCR, G protein–coupled receptor; LTCC, L-type calcium channel; mTORC1, mammalian target of rapamycin complex 1; NFAT, nuclear factor of activated T cells; NP, natriuretic peptide; PLB, phospholamban; RGS, regulator of G protein signalling; RyR2, ryanodine receptor; SERCA, sarcoplasmic/endoplasmic reticulum calcium ATPase; SR, sarcoplasmic reticulum; TRPC, transient receptor potential cation channel; TSC2, tuberin

Cardiac cAMP synthesis is catalysed by adenylyl cyclases (ACs) principally in response to beta 1- and beta 2-adrenergic receptor (β_1/2_-AR) stimulation by noradrenaline (NA) [5]. Within the heart, cAMP signals primarily by way of cAMP-dependent protein kinase (PKA) and exchange protein activated by cAMP (EPAC, a guanine nucleotide exchange factor that binds cAMP) [6]. PKA provokes positive chronotropic (i.e. heart rate), inotropic (i.e. contractile force), lusitropic (i.e. diastolic relaxant) and dromotropic (i.e. atrioventricular node depolarisation) cardiac responses by phosphorylating a number of cardiomyocyte-localised proteins. L-type calcium (Ca^2+^) channel (LTCC) phosphorylation raises the L-type Ca^2+^ current (*I*_Ca,L_) [7], and phosphorylation of the sarcoplasmic reticulum (SR) ryanodine receptor (RyR2) [8] increases further the intracellular concentration of Ca^2+^ ([Ca^2+^]_i_; although controversy persists regarding the role of RyR2 phosphorylation in cardiac physiology and HF pathophysiology) [9, 10]. Ca^2+^ reuptake is simultaneously promoted via phospholamban (PLB) phosphorylation and sarcoplasmic/endoplasmic reticulum Ca^2+^ ATPase (SERCA) activation [11]. Additionally, PKA regulates several cardiac excitation-contraction–coupling (ECC) proteins including cardiac troponin I (cTnI) [12], titin [13] and cardiac myosin–binding protein C (cMYBPC) (Fig. 1) [14].

Production of cGMP is initiated by guanylyl cyclases (GCs), which are sensitive to either nitric oxide (NO) or natriuretic peptides (NPs). NO activates the largely cytoplasmic or soluble form of GC, denoted GC-1 and GC-2 (formerly termed sGC) [15]. Conversely, the NPs stimulate the transmembrane or particulate GC, with atrial and brain NPs (ANP and BNP, respectively) activating GC-A, whilst C-type NP (CNP) binds GC-B [15]. cGMP-dependent protein kinase (PKG) is the predominant effector of cGMP signalling in the heart. PKG-facilitated phosphorylation modulates PLB [16], as well as myofilament components, for instance cTnI [17], troponin-t [18], titin [19] and cMYBPC [20]. PKG inhibits the transient receptor potential cation channel 6 (TRPC6) to counteract the calcineurin-nuclear factor of activated T cells (NFAT) pathway [21, 22] and activates the regulator of G protein signalling subtype 4 (RGS4) to offset G_i/q_ activation [23]. Hence, PKG is anti-adrenergic. PKG also phosphorylates tuberin (TSC2), restraining mammalian target of rapamycin complex 1 (mTORC1) activity [24]. In this way, PKG regulates basal cardiac contractility, lowering inotropy and raising lusitropy, and precludes cardiac hypertrophy, fibrosis and apoptosis.

## Cyclic Nucleotides in Heart Failure Pathophysiology

Heart failure develops from an initial insult to the myocardium. Cardiomyocyte death following a myocardial infarction (MI) [25], pressure overload during hypertension (HT) [26] and doxorubicin-induced cardiotoxicity [27] are among the most common causes of myocardial damage. The sympathetic nervous system (SNS) responds by stimulating chronotropy, inotropy and lusitropy in a cAMP/PKA-dependent manner. Initially, this conserves cardiac function, sustaining a CO and blood pressure necessary to uphold organ perfusion. However, continual cAMP/PKA signalling elicits maladaptive remodelling, comprising left ventricular hypertrophy (LVH), cardiac fibrosis and cardiomyocyte apoptosis [28–31]. In contrast, cGMP/PKG mitigates the injurious effects of repeated cardiac sympathetic activation, precluding the hypertrophy and fibrosis engendered by NA and angiotensin II (AngII, a central component of the renin-angiotensin-aldosterone system, which is also upregulated in HF) [32, 33]. cGMP also limits adverse remodelling in the wake of ischaemia-reperfusion injury (IRI) [34–36] and guards against arrhythmia induction in the wake of infarction [37]. NO bioavailability and NP signalling are impaired in HF patients, despite greater circulating NP concentrations, which heightens hospitalisation and mortality [38–42], whereas greater plasma concentrations of NA are associated with increased mortality risk in patients with HF [43].

## Cardiac Cyclic Nucleotide Phosphodiesterases

**Fig. 2:**
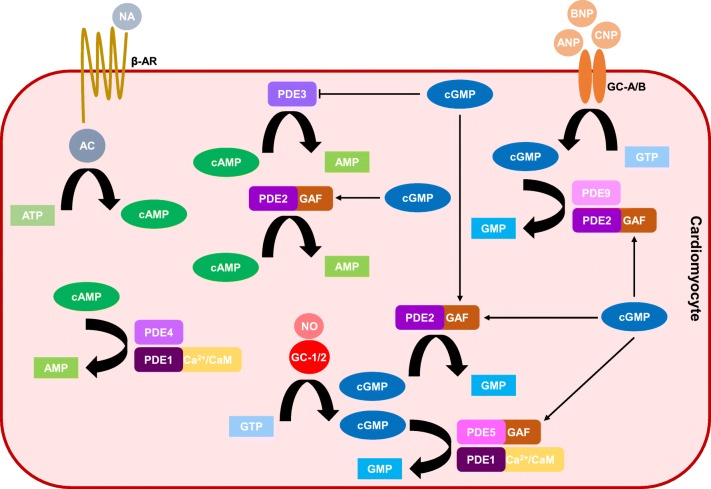
Phosphodiesterase regulation of cyclic nucleotides within cardiomyocytes. Phosphodiesterases (PDEs) catalyse the hydrolysis and inactivation of cyclic adenosine-3′,5′-monophosphate (cAMP) and cyclic guanosine-3′,5′-monophosphate (cGMP) forming adenosine monophosphate (AMP) and guanosine monophosphate (GMP), respectively. PDEs 1, 2, 3, 4, 5 and 9 are the principal regulators of cAMP and cGMP signalling in cardiomyocytes. PDE1 is calcium/calmodulin (Ca^2+^/CaM)-dependent and hydrolyses cAMP and cGMP. PDE2 is stimulated by cGMP binding to its GAF-B domain. PDE2 degrades cAMP and cGMP, and there is evidence that PDE2 hydrolyses both the natriuretic peptide (NP) and the nitric oxide (NO) pools of cGMP. PDE3 is a dual esterase but is inhibited by cGMP, and PDE4 is cAMP-selective. PDE5 is stimulated by cGMP binding to its GAF-A domain and largely hydrolyses NO/cGMP. PDE9 metabolises NP/cGMP. Abbreviations: AC, adenylyl cyclase; ANP, atrial natriuretic peptide; ATP, adenosine-5′-triphosphate; β-AR, beta-adrenergic receptor; BNP, brain natriuretic peptide; CNP, C-type natriuretic peptide; GAF, cGMP-stimulated PDE, *Anabaena* AC and Fhla transcription factor; GC, guanylyl cyclase; GTP, guanosine-5′-triphosphate; NA, noradrenaline

The PDE superfamily is made up of eleven closely related isozymes (PDE1–11) that are categorised according to the homology of the amino acid sequence within their C-terminal catalytic domains and distinguished by variations in their N-terminal regulatory regions [44]. Each isozyme is further classified into subtypes (gene products), of which multiple isoforms (splice variants) may exist. All PDEs catalyse the hydrolysis and inactivation of the 3′-cyclic phosphodiester bond of cAMP and/or cGMP. Within the heart, PDEs 1, 2, 3, 4, 5, 8 and 9 are expressed. Because PDEs are the prime regulators of cyclic nucleotides, they are responsible for integrating the often disparate signalling cascades of cAMP and cGMP in the heart (Fig. 2). Moreover, by controlling when and where hydrolysis occurs, PDEs confine cAMP and cGMP to separate subcellular compartments, compartmentalising individual cardiac cyclic nucleotide pools [45]. In view of this, it is not surprising that changes in PDE expression and/or activity are liable, somewhat, for the alterations in cAMP and cGMP during LVH and HF.

## Phosphodiesterase 1

### Overview

**Fig. 3:**
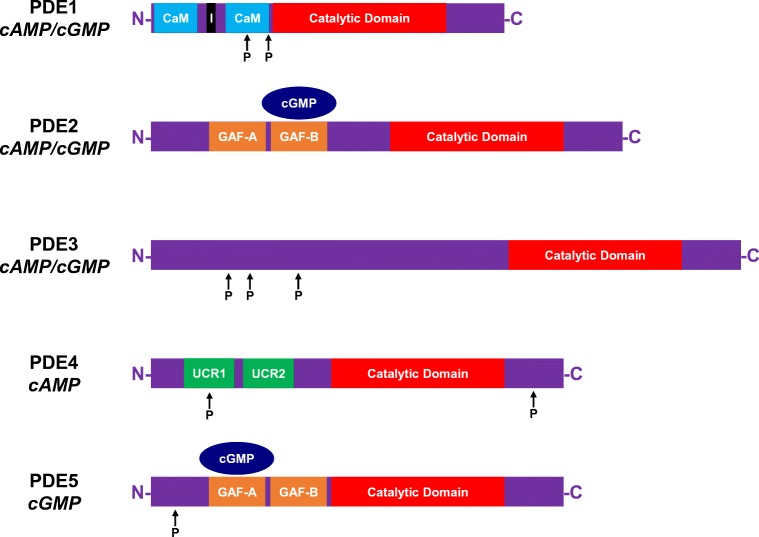
Structures of phosphodiesterases 1–5. Phosphodiesterase (PDE) 1 contains two calmodulin (CaM) binding domains, two phosphorylation sites and an inhibitory (I) region. PDE2 and PDE5 possess two cGMP-stimulated PDE, *Anabaena* AC and Fhla transcription factor (GAF) domains. Binding of cyclic guanosine-3′,5′-monophosphate (cGMP) to GAF-B and GAF-A stimulates the hydrolytic activity of PDE2 and PDE5, respectively. PDE3 can be phosphorylated at multiple regions, and PDE4 contains one phosphorylation site within its first upstream conserved regions (UCRs). Abbreviations: C, carboxyl-terminus; N, amino terminus; P, phosphate

Members of the PDE1 isozyme family are Ca^2+^/calmodulin (CaM)-dependent enzymes. Each subtype (PDE1A, PDE1B, PDE1C) contains at their N-termini two CaM binding domains, two phosphorylation sites and an inhibitory region that maintains the protein in an inactive configuration when the [Ca^2+^]_i_ is low (Fig. 3) [46]. Phosphorylation of PDE1 by either PKA (for PDE1A and PDE1C) [47, 48] or Ca^2+^/CaM-dependent protein kinase II (CaMKII; for PDE1B) [49] reduces the affinity of each subtype for Ca^2+^/CaM, thereby limiting enzymatic activity. Conversely, the binding of CaM to its respective sites elevates hydrolytic activity by preventing PKA/CaMKII-mediated phosphorylation, as well as effecting a conformational change that raises the maximal catalytic activity (*V*_max_) [50, 51]. PDE1 functions as a dual esterase, hydrolysing cAMP and cGMP. Whilst PDE1C hydrolyses each cyclic nucleotide with comparably high affinity (low Michaelis constant, *K*_M_), PDE1A and PDE1B display a lower affinity for cAMP, hence favouring cGMP hydrolysis [52, 53]. (Readers interested in exploring the enzymology of PDEs further are directed to the following excellent reviews) [44, 54].

### Cardiac Physiology

Both PDE1A and PDE1C messenger RNAs (mRNAs) are present in the human heart [53], with PDE1C serving as the principal subtype [55]. Although the majority of cardiac cyclic nucleotide hydrolysis is mediated by PDE1 in humans [56, 57], its roles in cardiac physiology are largely unknown. PDE1C is transcriptionally regulated by peroxisome proliferator–activated receptor alpha (PPARα) [58]. In cardiomyocytes, PDE1C shows a predominantly cytosolic distribution, localising to the M- and Z-lines of the sarcomere, and is present in microsomal fractions [55]. PDE1A protein is abundant in rabbit sinoatrial (SA) node cells where it is purported to moderate pacemaker activity [59], but whether it functions in an analogous capacity in human hearts is currently unknown. Similarly, whilst PDE1A appears to regulate cell death in vascular smooth muscle cells (VSMCs) [60], a corresponding cardiac-specific role is not established.

### Heart Failure Pathophysiology

Phosphodiesterase 1C mRNA and protein are raised in failing mouse and human hearts [61]. Likewise, PDE1A protein expression is increased by AngII and the β-AR agonist isoprenaline (ISO) in isolated cardiomyocytes, as well as following pressure overload (i.e. transverse aortic constriction, TAC) in vivo [62]. cAMP/PKA signalling is maintained in PDE1C^−/−^ cardiomyocytes, which moderates AngII- and ISO-stimulated hypertrophy and apoptosis, and PDE1C^−/−^ mice exhibit an improved phenotype with TAC relative to wild-type (WT) animals [61]. AngII promotes PDE1A levels in isolated rat cardiac myofibroblasts, and PDE1 inhibition (PDE1i) ameliorates the cardiac fibrosis associated with ISO-induced HF via cAMP and cGMP [63]. Although PDE1C is absent from cardiac fibroblasts, PDE1C deletion is anti-fibrotic, which may be a consequence of either diminished cardiomyocyte apoptosis or enhanced protective signalling between the two cell types [61]. Although this remains unclear, multidrug-resistant proteins (MRPs) have been implicated in the efflux of cAMP and cGMP [64, 65]. This could account for intercellular cyclic nucleotide signalling, and MRPs constitute prospective drug targets in HF. The hypertrophic and fibrotic actions of AngII are blunted by the PDE1 inhibitor vinpocetine in vitro and in vivo [66], and PDE1i improves cardiac function in failing mouse hearts through greater proteasomal activity [67]. Indeed, the pharmacological and genetic ablation of PDE1 was recently shown to enhance cAMP signalling through the adenosine A_2_ receptor (A_2_R), which is protective in multiple models of HF (including in larger mammals, e.g. rabbits and dogs), enhancing inotropy and vasodilation, as well as limiting apoptosis [68, 69]. It has been proposed that targeting the A_2_R, rather than the β-AR, pool of cAMP will circumvent the unfavourable actions of positive inotropic agents in HF (see below). However, this awaits further verification, as do the precise effects of raising this particular cAMP pool on hypertrophy and fibrosis. The upcoming clinical trial examining the safety and tolerability of the PDE1 inhibitor, ITI-214, in HF patients will undoubtedly illuminate these issues further (Clinicaltrials.gov: NCT03387215).

## Phosphodiesterase 2

### Overview

Like PDE1, PDE2 is capable of hydrolysing both cAMP and cGMP (Fig. 2), displaying comparable maximal rates and low *K*_M_ values for each cyclic nucleotide (with a slight preference for cGMP) [70, 71]. PDE2 is commonly designated as the *cGMP-stimulated* PDE since the enzyme possesses a regulatory segment at its N-terminus comprising two cGMP-stimulated PDE, *Anabaena* AC and Fhla transcription factor (GAF) domains (Fig. 3). Designated GAF-A and GAF-B, these domains are so called after the proteins in which they were originally discovered [72]. cGMP selectively binds to the allosteric GAF-B domain with high affinity, altering the conformation of PDE2 and raising esterase activity by a factor of 30 [73, 74]. It is unlikely that cAMP regulates PDE2 activity in vivo in the same way given that the affinity of cAMP for the GAF-B site is approximately 100-fold lower than cGMP [75]. A single isogene product of PDE2 (PDE2A) exists, and alternative gene splicing gives rise to three distinct isoforms that are either soluble (PDE2A1) or particulate (PDE2A2, PDE2A3) in their distribution [76, 77].

### Cardiac Physiology

Phosphodiesterase 2 is expressed in human hearts [78] and associates with the sarcomere and the plasma membrane in isolated cardiomyocytes [79]. As is the case for the majority of investigations pertaining to PDE expression and function, much of our knowledge concerning PDE2 has been obtained from in vitro analyses in isolated cardiomyocytes. Though there are evident shortcomings of such techniques (e.g. disparities between neonatal and adult cells, complications in integrating in vitro and in vivo physiology), PDE2 is increasingly recognised as an important moderator of cardiac function despite accounting for only a minor fraction of total cardiac PDE activity [80]. Indeed, given its activation by cGMP and its dual substrates, PDE2 occupies a unique position; not only does it facilitate negative feedback of cGMP signalling, it also mediates cross-communication between the cGMP and cAMP pathways. cGMP-induced activation of PDE2 reduces the *I*_Ca,L_ following greater cAMP hydrolysis and diminished LTCC phosphorylation by PKA [81–84]. PDE2 also contributes to the control of cardiac contractility by opposing the effects of β_1/2_-AR activation through cAMP degradation [79]. This appears largely dependent upon activation by NO/cGMP and β_3_-AR signalling, which can generate NO via NO synthase 3 (NOS3; formerly termed endothelial NO synthase/eNOS) stimulation [85]. Although β_3_-AR expression within the myocardium is contentious [86], this corresponds well with the different actions attributed to NO/cGMP and NP/cGMP on contractility. That is, whilst NO acts, primarily, as a negative inotrope, ANP either reduces or has no effect on inotropy [87, 88]. PDE2 also promotes cardiomyocyte apoptosis [89] and is highly expressed in cardiac fibroblasts where it regulates myofibroblast formation and fibrosis by counteracting the increase in cAMP in response to ISO and β-AR stimulation [90, 91].

Despite sharing a downstream second messenger, NO and NPs exert distinctive effects in both the heart and the vasculature. PDE2 makes a significant contribution to this by compartmentalising the cGMP signal in discrete cells and distinct intracellular regions. For example, in isolated rat cardiomyocytes, NO promotes the synthesis of a cytoplasmic pool of cGMP that is hydrolysed specifically by PDE5, whereas NPs generate a separate juxta-membrane GMP pool that is regulated by PDE2 [92]. Similarly, PDE2 confines the membrane-associated pool of cGMP generated via NP/GC-A signalling within the region of the T-tubules in isolated cardiomyocytes [93]. However, the role of PDE2 in restricting the sphere of activity of NP/cGMP can change with disease [87].

### Heart Failure Pathophysiology

Although the activity of myocardial PDE2 is amplified in pre-clinical models of pressure overload and in human HF [94, 95], its precise role in HF pathogenesis remains controversial. During HF, desensitisation of β-AR signalling occurs following prolonged SNS activation. Indeed, in ventricular cardiomyocytes from failing human hearts, β_1_-AR mRNA and protein expression are severely diminished relative to non-failing hearts, whereas those of β_3_-AR are tripled [96]. Consequently, the positive inotropic actions of β_1_-AR stimulation are hampered, whereas the negative inotropic effects of β_3_-AR signalling are maintained. Whether this is beneficial or detrimental to the failing heart is unclear, and PDE2 may be protective or harmful depending on this context. Firstly, cardiomyocyte-specific overexpression of PDE2 lowers the [Ca^2+^]_i_ and the *I*_Ca,L_, guarding against catecholamine-induced hypertrophy and inotropy in vitro [95]. Similarly, mice overexpressing PDE2 are resistant to arrhythmias and their cardiac contractility is maintained following MI [97]. Thus, PDE2 upregulation may well defend against sustained SNS signalling, and its activation could offer therapeutic benefit in HF. On the other hand, since PDE2 activation via the β_3_-AR/NOS3/NO/cGMP pathway abrogates β-AR-induced cAMP in cardiomyocytes and PDE2i can at least moderately restore β-AR responsiveness, PDE2 signalling might be unfavourable in HF [79]. Thus, inhibition of PDE2 could benefit the failing heart by restoring systolic function; recent work has given credence to this concept.

Whilst the inhibition of PDE3 and PDE4 elevates the concentration of cAMP and fosters hypertrophy in isolated cardiomyocytes, PDE2i is anti-hypertrophic [98]. This is thought to occur via an increase in a confined cAMP pool that promotes PKA activation, limiting pro-hypertrophic NFAT signalling. Likewise, PDE2i curtails the development of LVH, compromised contractility and cardiac fibrosis in pressure overload–induced HF. Rather than amplifying cAMP signalling, inhibition of PDE2 augments cGMP signalling, which is dependent upon endogenous NO activity and stimulation of GC-1 (rather than NP-activated GC-A) [87]. This supports previous suggestions that increases in cGMP produced by PDE2i functions akin to cAMP to curtail fibroblast to myofibroblast conversion and restrain fibrosis [91]. Pharmacological blockade of PDE2 also exerts favourable effects in mice with right ventricular hypertrophy (RVH) and pulmonary HT (PH); although in the right side of the heart, GC-A, rather than GC-1, plays an obligatory role in mediating the actions of PDE2i [99].

These disparate observations regarding PDE2 in HF pathology are likely attributable to differences in the concentrations of cyclic nucleotide generated in the varying models, as well as the different cyclic nucleotide pools that PDE2 regulates. Indeed, it has been postulated that PDE2 favours cGMP hydrolysis when NO signalling is diminished, whilst biasing toward cAMP hydrolysis in the setting of intense β-AR activation [100]. Whilst there are always difficulties translating pre-clinical observations into patients, the insights offered from the studies of PDE2 also afford the potential of stratifying treatment and targeting PDE2 selectively, depending on the nature of pathology. For example, given the positive pharmacodynamic profile of PDE2i during pressure overload, as well as the recent observations that blocking PDE2 activity specifically within cardiac sympathetic neurons may be advantageous during sympathetic hyperactivity [101, 102], inhibiting PDE2 may be effective in the context of HF and HT. Alternatively, enhancing PDE2 activity could perhaps help the failing heart post-MI. Further study of PDE2 as a therapeutic target in HF is certainly warranted.

## Phosphodiesterase 3

### Overview

Phosphodiesterase 3 is the third dual esterase. It hydrolyses cAMP and cGMP with high affinity, but the *V*_max_ for cGMP is ten times lower than that for cAMP [71]. That is to say, whilst cGMP binds to the catalytic site of PDE3, it is hydrolysed very slowly, which lessens cAMP metabolism. Hence, contrary to PDE2, PDE3 is often termed the *cGMP-inhibited* PDE (Fig. 2). This positions PDE3 as an important regulator of cAMP/cGMP crosstalk as cGMP serves as a positive regulator of cAMP signalling through PDE3 [103, 104]. However, PDE3 also possess three phosphorylation sites at its N-terminus, and PKA negatively modulates its own signal by phosphorylating PDE3, which raises enzymatic activity and cAMP hydrolysis (Fig. 3) [105, 106]. The two subtypes of PDE3 (PDE3A, PDE3B) are both present in the heart, but PDE3A predominates in the myocardium where (in conjunction with PDE4) it accounts for the majority of cAMP hydrolytic activity [107–109].

### Cardiac Physiology

By kerbing cAMP signalling, PDE3 is a key regulator of cardiac contractility. PDE3A associates with intracellular membranes in isolated rat cardiomyocytes and localises to Z-bands in human cardiomyocytes where it forms a scaffold with SERCA and PLB at the SR [80, 110]. PKA-mediated phosphorylation of PDE3 potentiates this interaction, and the degradation of cAMP precludes PLB phosphorylation by PKA and SERCA activation. Hence, PDE3 controls Ca^2+^ reuptake into the SR [110, 111]. PDE3 also limits contractility by curtailing the *I*_Ca,L_ [112, 113]. PDE3A is largely responsible for this as PDE3A^−/−^ mice, but not PDE3B^−/−^ animals, exhibit increased chronotropy basally compared to WT [114]. Likewise, the inotropic and chronotropic responses to ISO are not potentiated by the PDE3 inhibitor cilostamide in PDE3A^−/−^ mice, but these are in PDE3B^−/−^ animals. PDE3 also reduces spontaneous SA node activity by restricting cAMP-mediated activation of hyperpolarisation-activated cyclic nucleotide (HCN)–activated channels, as well as by reducing the phosphorylation of several pacemaker components (e.g. LTCC, RyR2, PLB) by PKA [115–117]. PDE3B complexes with phosphatidylinositol 3-kinase-gamma (*PI3Kγ*) in the mouse myocardium, which stimulates PDE3B activity, reducing cAMP levels and cardiac contractility [118]. PDE3B also interacts with *PI3Kγ* in human arterial endothelial cells and increases endothelial cell proliferation and angiogenesis through cAMP degradation and diminution of PKA [119, 120]. Whether a similar phenomenon occurs within the myocardium is unknown but seems probable given the associations observed in mouse hearts. Additionally, PDE3A reduces cardiomyocyte apoptosis by preventing inducible cAMP early repressor (ICER) expression, which stops the reduction in pro-survival genes [121, 122].

Phosphodiesterase 3 is differentially regulated by NO/cGMP and NP/cGMP. GC-1^−/−^ mice exhibit impaired PDE3 expression and activity [123]. Although NO/cGMP is predominantly negatively inotropic, low concentrations of NO/cGMP can inhibit PDE3 [124]. Whilst this increases a particular cAMP/PKA pool in isolated cardiomyocytes, ANP/cGMP favours activation of PDE2, which decreases cAMP and PKA activation [88]. Thus, whilst NO/cGMP can exert dual effects on cardiac contractility, ANP/cGMP tends to oppose inotropy. CNP/cGMP, however, boosts the positive inotropic and lusitropic responses to β-AR stimulation in isolated rat cardiomyocytes via PDE3 antagonism, whilst BNP/cGMP does not [125]. Therefore, PDE3 is also distinctively controlled by different NPs.

### Heart Failure Pathophysiology

Both the expression and activity of PDE3A are diminished in pre-clinical models of pressure overload and in failing human hearts [121, 126]. Pharmacological inhibition of PDE3 has been reported to blunt TAC-stimulated hypertrophy and fibrosis, an effect recapitulated in PDE3A^−/−^, but not PDE3B^−/−^, mice [127]. Conversely, genetic ablation of PDE3B, but not PDE3A, counteracts the adverse effects of IRI [128]. This occurs following PKA-mediated opening of mitochondrial Ca^2+^-activated potassium (K^+^) channels, which reduces reactive oxygen species formation and protects against apoptosis. Although such findings intimate that PDE3 downregulation could represent a protective mechanism in HF and that selective targeting of PDE3 may provide therapeutic benefit to failing hearts, this is clearly not the case. The diminution of PDE3 activity is associated with β-AR desensitisation in LVH, and PDE3i fosters cardiomyocyte apoptosis [121, 126]. Similarly, greater PDE3A expression protects against IRI and apoptosis in the murine heart by regulating β-AR/cAMP [129, 130], whilst the formation of a complex between PDE3B and *PI3Kγ* guards against TAC-induced cardiac dysfunction [118].

Similar detrimental outcomes have been observed in patients. It was originally postulated that inotropes would be beneficial in HF. Indeed, the PDE3 inhibitor milrinone acutely increases inotropy, chronotropy and stroke volume, whilst reducing MABP in HF patients [131]. However, prolonged PDE3i is not beneficial to the failing heart since it exacerbates SNS activity, increasing arrhythmias and mortality [132–135]. This may be accounted for by the adverse effects discussed above regarding PDE3 blockade and could be attributable to genetic polymorphism within PDE3 in populations of HF patients [136]. It has been suggested that these side effects may be offset by more tailored therapy targeting particular PDE3 isoforms (milrinone is a non-specific PDE3 inhibitor) or by concomitant administration of PDE3 and β-AR antagonists. Whilst such a tandem therapy is well tolerated in HF patients, it fails to improve exercise capacity and mortality [137]. Therefore, positive inotropic agents (including PDE3 inhibitors and β-agonists) are now only used to improve haemodynamic status in acute decompensated HF or as a bridge to heart transplantation [137, 138]. Milrinone may provide some benefit in the context of HF with preserved ejection fraction (HFpEF) [139], and an extended release formulation exhibits early positive effects in advanced HF [140]. HFpEF may constitute as much as 70% of all HF cases [141, 142], and unlike those with HF with reduced ejection fraction (HFrEF), these patients, the majority of whom are older and female, do not respond as well to routine treatment (e.g. angiotensin-converting enzyme inhibitors) [143]. Presently, there is no licenced medication for HFpEF, and targeting PDE3 may afford a novel therapeutic paradigm. For mature women with HFpEF, this would likely be more efficacious than targeting PDE5, for example, since the menopause is associated with impaired NO bioactivity [144] and PDE5 inhibition fosters NO/cGMP signalling specifically (see below). However, larger, longer-term randomised clinical evaluation of PDE3 blockade in HFpEF is necessary.

## Phosphodiesterase 4

### Overview

The four subtypes of PDE4 (PDE4A, PDE4B, PDE4C, PDE4D) are highly selective for cAMP, displaying very low *K*_M_ values for this cyclic nucleotide [145–147]. Alternative gene splicing produces more than twenty PDE4 isoforms, which can exist in *short* and *long* forms depending on the presence of so-called upstream conserved regions (UCRs) within the N-terminus (Fig. 3) [148]. Short PDE4 isoforms possess a single UCR (UCR2), whilst long versions contain two (UCR1, UCR2). PDE4 possesses a phosphorylation site within UCR1, and PKA-mediated phosphorylation elevates esterase activity by precluding UCR1-UCR2 interactions [149]. An extracellular signal–regulated kinase (ERK) phosphorylation region at the C-terminus serves as a negative and positive regulator of long and short isoforms, respectfully [150].

### Cardiac Physiology

In the heart, PDE4A, PDE4B and PDE4D are expressed whereas PDE4C is not [151]. In mouse and rat cardiomyocytes, PDE4 behaves analogously to PDE3 and perpetuates the main cAMP hydrolytic activity (although PDE4 constitutes a substantially smaller fraction of total PDE activity in human hearts) [152]. PDE4 and PDE3 are the principal PDEs adjusting cAMP levels and *I*_Ca,L_ basally, whilst PDE4 predominantly contributes to the control of β-AR–stimulated elevations in cAMP, *I*_Ca,L_ and cardiac contractility [80, 113]. Indeed, PDE4 is capable of interacting with β_1/2_-ARs and the LTCC, and the *I*_Ca,L_ is amplified in PDE4^−/−^ cardiomyocytes in response to ISO [153–155]. Additionally, PDE4 associates with RyR2 and PLB/SERCA [156–158]. By curtailing cAMP at the SR, PDE4 limits PKA-induced phosphorylation of these sites, reducing Ca^2+^ release/reuptake. Like PDE3, PDE4 also regulates the pacemaker activity of the SA node [117, 159, 160].

### Heart Failure Pathophysiology

Resembling PDE3A, the expression and activity of PDE4A and PDE4B, but not PDE4D, declines with pressure overload in rats as well as in human HF [126]. Consequently, the control of β-AR–stimulated cAMP by PDE4 is diminished. Moreover, when PDE4 is knocked out, the heart is rendered more susceptible to ventricular tachycardia in response to β-AR agonists, and arrhythmogenesis is exacerbated when PDE4 is inhibited [155, 159, 161, 162]. PDE4^−/−^ mice experience more severe HF with MI due to heightened RyR2 phosphorylation and defective Ca^2+^ regulation [156]. This likely contributes to the cardiac dysfunction in failing human hearts, also [8].

## Phosphodiesterase 5

### Overview

Phosphodiesterase 5 is both selective for and activated by cGMP. Akin to PDE2, PDE5 contains GAF-A and GAF-B domains within its N-terminus, and its esterase activity is promoted by cGMP binding to the GAF-A site (Fig. 3) [163–165]. PKG-mediated phosphorylation of PDE5 amplifies the cGMP affinity of the GAF-A domain, thereby stabilising the active enzyme conformation and maintaining hydrolytic activity [166–168]. Thus, cGMP fosters its own degradation through negative feedback (i.e. facilitated hydrolysis).

### Cardiac Physiology

The one PDE5 subtype (PDE5A) is alternatively expressed as three isoforms (PDE5A1, PDE5A2, PDE5A3), although dispute persists concerning the physiological relevance of these to cardiac physiology. Whilst some studies have reported either no or minimal cardiac expression of PDE5 [56, 169–171], others have detected it within certain cell types [172–174]. In isolated cardiomyocytes, PDE5 appears restricted to the cytosol where it is anchored to the Z-lines and preferentially hydrolyses NO/cGMP (although this may change with disease) [92, 175, 176]. Inhibition of PDE5 with sildenafil (Viagra) suppresses the contractile response to β-AR stimulation in isolated mouse cardiomyocytes and human hearts [175, 177]. This is dependent upon greater NOS3-derived cGMP and heightened PKG activity and can be enhanced by sGC stimulation [178, 179]. Sildenafil also enhances the cGMP-dependent activation of PDE2, which reduces chronotropy via attenuation of cAMP signalling [180]. PDE5 is expressed in cardiac fibroblasts and likely participates in cardiac fibroblast transformation and proliferation [170, 181]. In vascular endothelial cells, PDE5 localises to caveolae where it negatively modulates NOS3 signalling [182], and PDE5 is abundantly expressed in VSMCs where it plays a central role in regulating vascular tone [56, 183].

### Heart Failure Pathophysiology

Since PDE5 knockout models are still lacking, particularly those of a cell-restricted nature, knowledge of its precise physiological role in the heart remains elusive. However, it is clear that PDE5 contributes to the pathophysiology of HF. Greater levels of PDE5 are observed in multiple pre-clinical models of pressure overload and cardiac ischaemia [184, 185] (although exceptions have been reported) [170]. Moreover, PDE5 is upregulated in PH patients with RVH [186, 187] and during human LVH and HF [184, 188]. Blockade of PDE5 with sildenafil reduces infarct size and improves cardiac contractility and survival in mice with MI [189]. This is mediated by PKG, which diminishes cardiomyocyte apoptosis through the inhibition of glycogen synthase kinase 3 beta (GSK3β) [190] and protects the heart via hydrogen sulphide synthesis and the opening of mitochondrial ATP-sensitive K^+^ channels [191–193]. Sildenafil also bolsters the degradation of damaged proteins by the proteasome [194], which may further benefit the failing heart. Vardenafil and tadalafil, two further selective PDE5 inhibitors, display similar protective profiles to sildenafil. Vardenafil maintains diastolic function in rats with HFpEF associated with diabetic cardiomyopathy [195], and tadalafil attenuates doxorubicin-induced PKG oxidation and cardiac dysfunction in mice [196, 197]. Tadalafil has also been shown to promote contractility in experimental HF by raising the density of transverse tubules in cardiomyocytes, thereby improving β-AR responsiveness [198].

The anti-hypertrophic effects exerted by PDE5i appear dependent upon PKG. Sildenafil attenuates NFAT activation by amplifying PKG-mediated inactivation of TRPC6, which prevents and reverses LVH in mice after TAC [199–201]. Sildenafil also suppresses hypertrophy by promoting the activation of RGS2 by PKG [202]. These effects are lost in mice expressing a dysfunctional form of PKG [203], and the hypertrophic response of isolated cardiomyocytes to phenylephrine is lessened following PDE5 gene silencing [176]. However, the contribution of PDE5 and PKG to cardiac hypertrophy has been contested. PKG deletion within the myocardium does not alter basal cardiac function and hypertrophy, nor does it affect the progression of AngII-mediated LVH- or ISO-induced dysfunction [170, 204, 205]. Yet, the loss of PKG in mice does exacerbate pressure overload-induced HF [204, 206].

Evidently, further research is needed to resolve these conflicting observations. Variations in the cellular distribution of PKG and mechanisms to compensate for the cardiomyocyte-specific loss of PKG probably contribute, as may the differences in the type of HF model employed and the possible concurrent inhibition of PDE1 and PDE5 with high doses of sildenafil [57, 170]. Nevertheless, even if impaired PKG signalling within the myocardium does not innately influence cardiac hypertrophy, it might render the heart more susceptible to fibrosis. PDE5 may not be detectable in cardiomyocytes in the wake of pressure overload, but its expression in cardiac fibroblasts is elevated with TAC [170]. Moreover, the ability of NP/cGMP to moderate collagen production in isolated cardiac fibroblasts is supplemented by PDE5i [207], and sildenafil mitigates fibroblast activation and fibrosis associated with pressure overload in mice [181]. PKG deficiency also negates the anti-fibrotic effects of sildenafil [204, 205]. This evidence establishes that PDE5 can be targeted pharmacologically for therapeutic gain in HF, even if its expression in cardiomyocytes per se is questionable.

Sildenafil lowers systolic pulmonary artery pressure (PAP) in HF patients [208, 209] and increases CO and reduces hospitalisations in HF patients with secondary pulmonary arterial HT (PAH) [210]. Though sildenafil causes a modest (approximately 10%) reduction in mean PAP in PAH patients [210], the salutary effects of PDE5 inhibition appear to occur through additional protection of the RV (e.g. reductions in RVH) [211]. The effects of PDE5 blockade on cardiac function in HFrEF are variable with both positive and negative effects on cardiac function observed [212–214]. Despite displaying early positive signs in HFpEF [215], sildenafil does not significantly improve cardiac structure and function or clinical status in these patients [216–219].

### Other Phosphodiesterases

Phosphodiesterases 6 and 11 are not found in the heart [220, 221]. PDE7 mRNA is detectable within the heart [222], but a cardiac-specific role for this PDE has not yet been recognised. PDE8 mRNA and protein are expressed in cardiomyocytes, and this cAMP-selective esterase might play a part in cardiac excitation-contraction coupling [223]. PDE10 has been implicated in RVH, yet this is presumably due to elevated expression within the pulmonary vasculature during PH [224]. PDE9 is surfacing as a decisive regulator of cGMP signalling. Like PDE5, it is a cGMP-specific esterase, though myocardial PDE9 curtails NP-mediated increases in cGMP rather than hydrolysing the cGMP pool generated by NO/GC-1 [225]. PDE9 is also expressed in VSMCs where, contrary to the myocardium, it was recently shown to metabolise NO/cGMP [226]. PDE9 is upregulated during LVH and HF in mice and humans, and its inhibition slows the progression of experimental HF [225, 227] by augmenting NP/cGMP-triggered pathways that are well established to be anti-hypertrophic and anti-fibrotic [228–230].

## Conclusion

**Fig. 4:**
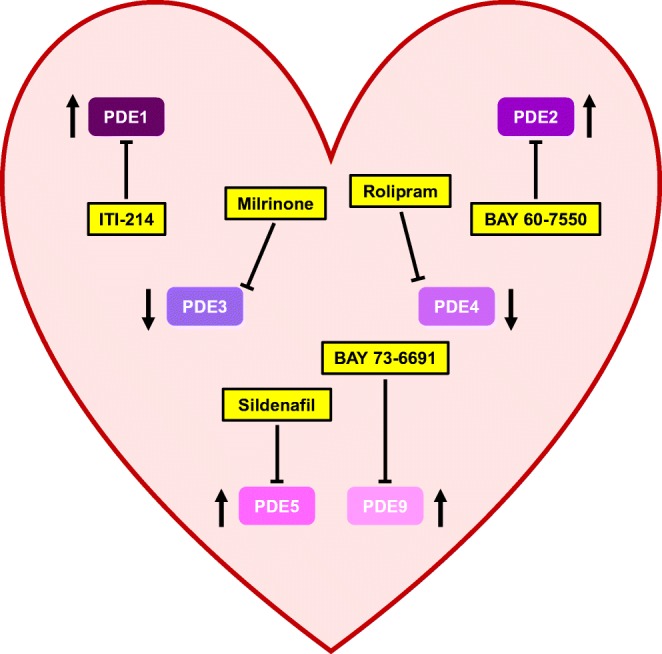
Changes in cardiac phosphodiesterase expression during heart failure and pharmacological modulators. Phosphodiesterases (PDEs) 1, 2, 3, 4, 5 and 9 contribute to the pathophysiology of heart failure (HF). Arrows indicate changes in PDE expression during HF. Blunt lines indicate inhibition with selective pharmacological modulators

The evidence discussed here demonstrates that PDEs occupy a pivotal position in cardiac physiology and HF pathophysiology. PDE expression changes in failing hearts and pharmacological manipulation of PDE activity ought to amend the aberrant cardioprotective cyclic nucleotide signalling characteristic of cardiac dysfunction (Fig. 4). Future research will aid in the identification of the interventions that will be most successful in realising the necessary, and often divergent, therapeutic objectives in the distinct types of HF. For example, PDE3 inhibition, whilst deleterious in HFrEF, may represent an effective means of treating HFpEF patients. Moreover, investigations into different combination therapies, such as the inhibition of multiple PDEs, or simultaneously activating GC (e.g. sGC stimulators) and blocking PDEs, should also prove fruitful. In sum, targeting PDEs will doubtless prove essential in rectifying the present dearth of effective HF therapies, thereby curtailing the morbidity and mortality associated with this disease.

## Abbreviations

[Ca^2+^]_i_,: Intracellular concentration of calcium
A_2_R: Adenosine A_2_ receptor
AC: Adenylyl cyclase
AngII: Angiotensin II
ANP: Atrial natriuretic peptide
β-AR: Beta-adrenergic receptor
BNP: Brain natriuretic peptide
Ca^2+^: Calcium
CaM: Calmodulin
CaMKII: Calcium/calmodulin-dependent protein kinase II cAMP Cyclic adenosine-3′-5′-monophosphate
cGMP: Cyclic guanosine-3′,5′-monophosphate
cMYBPC: Cardiac myosin–binding protein C
CNP: C-type natriuretic peptide CO Cardiac output
cTnI: Cardiac troponin I
ECC: Excitation-contraction coupling
EPAC: Exchange protein activated by cAMP
ERK: Extracellular signal–regulated kinase
GAF: cGMP-stimulated PDE, *Anabaena* AC and Fhla transcription factor
GC: Guanylyl cyclase
GSK3β: Glycogen synthase kinase 3 beta
HCN: Hyperpolarisation-activated cyclic nucleotide
HF: Heart failure
HFpEF: Heart failure with preserved ejection fraction
HFrEF: Heart failure with reduced ejection fraction
HT: Hypertension
*I*_Ca,L_: L-type calcium current
ICER: Inducible cAMP early repressor
IRI: Ischaemia-reperfusion injury
ISO: Isoprenaline
K^+^: Potassium
*K*_M_: Michaelis constant
LTCC: L-type calcium channel
LVH: Left ventricular hypertrophy
MI: Myocardial infarction
MRP: Multidrug-resistant protein
mTORC1: Mammalian target of rapamycin complex 1
NA: Noradrenaline
NFAT: Nuclear factor of activated T cells
NO: Nitric oxide
NOS: Nitric oxide synthase
NP: Natriuretic peptide
PAH: Pulmonary arterial hypertension
PAP: Pulmonary artery pressure
PH: Pulmonary hypertension
PDE: Phosphodiesterase
*PI3Kγ*: Phosphatidylinositol 3-kinase-gamma
PKA: cAMP-dependent protein kinase
PKG: cGMP-dependent protein kinase
PLB: Phospholamban
PPARα: Peroxisome proliferator–activated receptor alpha
RGS: Regulator of G protein signalling
RVH: Right ventricular hypertrophy
RyR2: Ryanodine receptor
SA: Sinoatrial
SERCA: Sarcoplasmic/endoplasmic reticulum calcium ATPase
sGC: Soluble guanylyl cyclase
SNS: Sympathetic nervous system
SR: Sarcoplasmic reticulum
TAC: Transverse aortic constriction
TRPC: Transient receptor potential cation channel
TSC2: Tuberin
UCR: Upstream conserved region
*V*_max_: Maximal catalytic activity
VSMC: Vascular smooth muscle cell
WT: Wild-type
